# Influenza and Pneumonia Vaccination Rates and Factors Affecting Vaccination among Patients with Chronic Obstructive Pulmonary Disease

**DOI:** 10.4274/balkanmedj.2016.1028

**Published:** 2017-05-15

**Authors:** Ülkü Aka Aktürk, Aslı Görek Dilektaşlı, Aysun Şengül, Banu Musaffa Salepçi, Nuray Oktay, Mustafa Düger, Hale Arık Taşyıkan, Nagihan Durmuş Koçak

**Affiliations:** 1 Clinic of Chest Diseases, Süreyyapaşa Chest Disease and Thoracic Surgery Training and Research Hospital, İstanbul, Turkey; 2 Departmen of Chest Diseases, Uludağ University School of Medicine, Bursa, Turkey; 3 Clinic of Chest Diseases, Derince Training and Research Hospital, Kocaeli, Turkey; 4 Clinic of Chest Diseases, Dr. Lütfi Kırdar Kartal Training and Research Hospital, İstanbul, Turkey; 5 Clinic of Chest Diseases, Erbağ State Hospital, Tokat, Turkey; 6 Clinic of Chest Diseases, Medipol Mega University Hospital, İstanbul, Turkey; 7 Department of Public Health, Yeditepe University School of Medicine, İstanbul, Turkey

**Keywords:** Chronic obstructive pulmonary disease, influenza vaccination, pneumonia vaccination, prevention

## Abstract

**Background:**

Influenza and pneumococcal vaccinations are recommended in chronic obstructive pulmonary disease patients to decrease associated risks at all stages. Although the prevalence of chronic obstructive pulmonary disease is high in our country, as previously reported, vaccination rates are low.

**Aims:**

To assess the vaccination rates of chronic obstructive pulmonary disease patients and factors that may affect these.

**Study Design:**

Multi-centre cross-sectional study.

**Methods:**

Patients admitted to the chest diseases clinics of six different centres between 1 February 2013 and 1 January 2014 with a pre-diagnosis of Chronic obstructive pulmonary disease according to the Global initiative for chronic obstructive lung disease criteria, who were in a stable condition were included in the study. The survey, which included demographic characteristics, socio-economic status, severity of disease and vaccination information, was first tested on a small patient population before the study. The survey was completed by the investigators after obtaining written informed consent.

**Results:**

The average age of the 296 included patients was 66.3±9.3 years and 91.9% were male. Of these, 36.5% had the influenza vaccination and 14.1% had the pneumococcal vaccination. The most common reason for not being vaccinated was ‘no recommendation by doctors’: 57.2% in the case of influenza vaccinations, and 46.8% in the case of pneumococcal vaccinations. Both vaccination rates were significantly higher in those patients with comorbidities (influenza vaccination p<0.001; pneumococcal vaccination p=0.06). There was no significant correlation with age, gender, smoking and severity of disease (p>0.05). Vaccination rates were significantly higher in those with a white-collar occupation and higher education level, and who presented to a university hospital (p<0.001).

**Conclusion:**

Medical professionals do not request vaccinations as often as the International Guidelines suggest for chronic obstructive pulmonary disease patients. Awareness of the importance of these vaccinations among both doctors and patients needs to be addressed.

The global incidence of Chronic obstructive pulmonary disease (COPD) is currently 11.8% among men and 8.5% among women. Recently, COPD was reported to be the fifth cause of death worldwide, and is expected to be the fourth by 2030 (1,2). COPD is a disease that progresses with each acute attack. The most frequent causes of these attacks are influenza and streptococcus pneumonia infections, which lead to increased hospitalization and mortality rates. The Global initiative for chronic obstructive lung disease (GOLD) recommends influenza vaccination at evidence A level due to its ability to reduce hospitalization and mortality rates, and pneumococcal vaccination at evidence B level to prevent society-based pneumonia (3).

Despite these recommendations, vaccination rates in patients with COPD are not at the desired level. The vaccination rates targeted by the American National Immunization Program Advisory for COPD patients are 90%. However, health statistics data show that the influenza vaccination rates in America between 1999 and 2005 were 70% at the most, and the pneumococcus vaccination rates were 49.9-56.3% (4). Another study conducted in Italy reported lower (30.5%) influenza vaccination rates among patients with COPD (5). According to the data obtained from the Burden of chronic obstructive lung disease study in our country, the prevalence of COPD was 15.4% among men and 6% among women (1). Several studies performed on an elderly population (>65 years) in Turkey indicated that the minimal influenza vaccination rate in patients with COPD was 5.9% and the maximal rate was 27.3% (6). In another single-centre study, pneumococcal and influenza vaccination rates were 51% (7).

There are a limited number of studies on vaccination rates among COPD patients and, of these, most are single- centre studies. We aimed to determine the influenza and pneumococcal vaccination rates in patients with COPD in our country, and the factors affecting these rates.

## MATERIALS AND METHODS

This was a multi-centre cross sectional study approved by the ethics committee of our university. The centres included were a university hospital, three training hospitals, and two public hospitals from five different cities in our country.

Patients admitted to the chest diseases clinics of six different centres between 1 February 2013 and 1 January 2014 with a pre-diagnosis of COPD according to the GOLD criteria (8), who were in a stable condition, and over 40 years of age, were included in the study. Patients not compliant with the respiratory function test (RFT), those who refused to participate in the study, pregnant patients, and patients with a cancer diagnosis were excluded from the study.

The survey, which included demographic characteristics, socio-economic status, severity of disease and vaccination information, was first tested on a small patient population before the study. The survey was completed by the investigators after obtaining written informed consent. The RFT technicians were trained prior to the study and spirometric tests were performed according to the standard criteria (9). The stages of the disease, Modified Medical Research Council (MMRC) and COPD Assessment Test (CAT) scores were determined according to GOLD (8,10).

### Statistical analysis

While measures subject to statistical analysis in the study were defined as the mean and standard deviation for continuous variables, they were defined as number and percentage values for categorical variables. The chi-square and Fisher’s exact probability tests were used to compare differences between the categorical variables. For a comparison of normally distributed continuous variables the t-test was used. A p value of <0.05 was considered significant. Statistical analyses were carried out using SPSS (Version: 21.0; IBM Corp., Armonk, NY, USA) package software.

## RESULTS

A total of 296 patients from six different centres who fulfilled the criteria were included in the study. Of these, 77.4% were admitted to public hospitals, and 22.6% were admitted to the university hospital. The mean age was 66.35±9.33, and 91.9% were male. A concomitant disease was present in 60.8% of cases (37% hypertension, 13.9% diabetes, and 23.1% cardiovascular diseases). According to GOLD, the patients were determined to have a stage 1 disease in 4.2%, stage 2 disease in 40.8%, stage 3 disease in 36.6%, and stage 4 disease in 18.5%. The mean CAT score was 21.65±8.63. According to the MMRC dysnoea scores, 9.5% were stage 1, 37% were stage 2, 27% were stage 3, and 18% were stage 4.

Of the 296 patients, 36.5% stated that they had received an influenza vaccination, and 14.1% a pneumococcal vaccination. Of these, 54% of the patients who had been vaccinated for influenza, and 38.1% who had been vaccinated for pneumococcus, had previously been recommended by the chest diseases physician to obtain the vaccination. The rates of physicians recommending vaccination, other than chest disease physicians, are presented in Table 1. The most notable reason for not being vaccinated for influenza or pneumococcus was ‘my doctor didn’t advise me to’ (57.2% and 46.8%, respectively). Other reasons are listed in Table 1.

When vaccinated patients were compared to those who were not vaccinated, the rate of concomitant disease was significantly higher in those patients who received the influenza vaccination (70.1%, 56.4%, respectively, p<0.001). The rate was also higher in the group who received the pneumococcal vaccination but this was not statistically significant (73.2%, 59%, respectively, p=0.06). No significant differences were observed between the other demographic characteristics between the vaccinated and non-vaccinated groups (p>0.05) (Table 2). No differences in the vaccination rates were observed according to the GOLD stages, CAT scores and MMRC scores (p>0.05). The vaccination rates were significantly higher in both vaccine groups among the white-collar workers and those with an education level of lycée or higher (p<0.001). No differences were observed with regard to the marital and economic status of the patients (Table 3). The influenza and pneumococcal vaccination rates were significantly higher in those patients who presented to the university hospital, compared to the public hospitals (50%, 32.8%, p<0.001; and 25.8%, 11%, p<0.001, respectively).

## DISCUSSION

In this study, the flu vaccination rate among COPD patients was 36.5%, and the pneumococcal vaccination rate was 14.1%. The most notable reason for non-vaccination was a lack of recommendation from the doctor. The most important indicators for increasing the rate of vaccination were the presence of a concomitant disease, level of education and occupation. Those with a lycée or higher educational degree and white-collar workers had statistically significantly higher vaccination rates.

International authorities recommend the flu vaccination at an evidence a level, due to its ability to reduce influenza-related attacks, hospitalization and mortality rates in patients with COPD. Pneumococcal vaccination, on the other hand, is recommended at an evidence B level, since it decreases society-based pneumonias and related attacks (3,8).

The worldwide-targeted vaccination rates for patients with COPD are high; however, studies show that the actual vaccination rates are much lower (4,5). A survey study by Chiatti et al. (5) in Italy, which included 6051 patients with COPD, showed the influenza vaccination rate to be 30.5%. Arinez-Fernandez et al. (11) determined the pneumococcal vaccination rate as 32.5%, and Vandenbos et al. (12) found the influenza vaccination rate to be 55.3% in patients with COPD. In Turkey, influenza vaccination rates in patients with COPD were found to be 27.3% and 14.9% in two different studies (6,13). In our study, we showed an influenza vaccination rate of 36.5% in patients with COPD, similar to rates previously reported in our country, and lower than those observed in American and European studies. The pneumococcal vaccination rate in our study was 14.1%, which is lower than that observed worldwide. We believe that the lack of pneumococcal vaccine reimbursement in our country contributes the lower rate in vaccination.

In a study by Chiatti et al. (5), the influenza vaccination rates were found to be higher in patients who had more frequent contact with their medical practitioners. On the other hand, in a Spanish study investigating pneumococcal vaccination rates and factors affecting these rates in patients with COPD, the number of general practitioner visits within the previous year did not affect the vaccination rates (11). In our study, the vaccination rates among patients with COPD were determined to be higher when recommended by a doctor, particularly a chest specialist. Furthermore, the lack of recommendation by a doctor was the most frequent cause of non- vaccination in our study.

In a study by Vandenbos et al. (12), the most important cause of non-vaccination for influenza was found to be refusal of vaccination or intolerance against vaccination, the most important cause of non-vaccination for pneumonia was found to be a lack of recommendation by the general practitioner. Overall, these results indicate the importance of the medical practitioner’s recommendation for vaccination.

The most important causes for non-vaccination in the study by Ciblak (6) were determined to be: not believing in the efficacy of the vaccines; believing that the vaccines trigger the flu; an unwillingness to repeat the dose; not believing themselves to be in the risk group; and worrying about the side-effects of the vaccine.

Other reasons for non-vaccination of the patients in our study were: unawareness of the usefulness of the vaccines or not believing it to be useful; not believing the necessity of the vaccines, and similar reasons. These observations indicate that patients with COPD in our country are not well informed on the efficacy of vaccinations.

There are conflicting reports in which some advocate that gender does not affect vaccination, while others claim that vaccination rates are higher among women (14,15,16,17,18). Likewise, some studies have concluded that being married positively affects the vaccination rate, while others conclude that marital status has no effect at all (5,18). Chiatti et al. (5) concluded that the influenza vaccination rates were higher among the elderly and those with concomitant disease, and lower among active smokers, single or divorced patients. In the same study, a high level of education among elderly patients with COPD was found to negatively affect the vaccination rate. In contrast, other studies indicated that higher educational levels improved the vaccination rates (18,19,20). In our study, gender, marital status, and smoking habits were found to have no effect on the pneumococcal or influenza vaccination rates. However, concomitant disease increased the influenza vaccination rates, although no statistically significant increase in pneumococcal vaccination rates was observed. Similar to that observed in the literature, the vaccination rates among COPD patients with an educational level of lycée or higher, those who had been admitted to the university hospital, or those who were white-collar workers, were found to be higher. Income status had no effect on either vaccination rates. According to the GOLD stages, CAT and MMRC scores that define clinical severity, no relationship was observed with the vaccination rates.

The strength of our study is that it is a multi-centre one that addresses the lack of data on vaccination rates among COPD patients in our country. The primary limitation of our study is that it was based on a survey and some of the patient statements may not be accurate.

In conclusion, influenza vaccination rate of patients with COPD in our country is lower than expected, and the pneumococcal vaccination rate is much lower than expected. The most notable cause of non-vaccination among patients was found to be a lack of recommendation by their doctor, and the second was the patients’ lack of knowledge regarding vaccinations. The higher vaccination rates among patients with concomitant diseases, those who had been admitted to the university hospital, and those with higher levels of education were most likely because they paid more visits to the doctor. These outcomes indicate the importance of the medical practitioner in informing the patient of the value of the vaccination and recommending that they receive it. In line with this, further training for doctors with respect to influenza and pneumococcal vaccinations in patients with COPD may be required.

## Figures and Tables

**Table 1:**
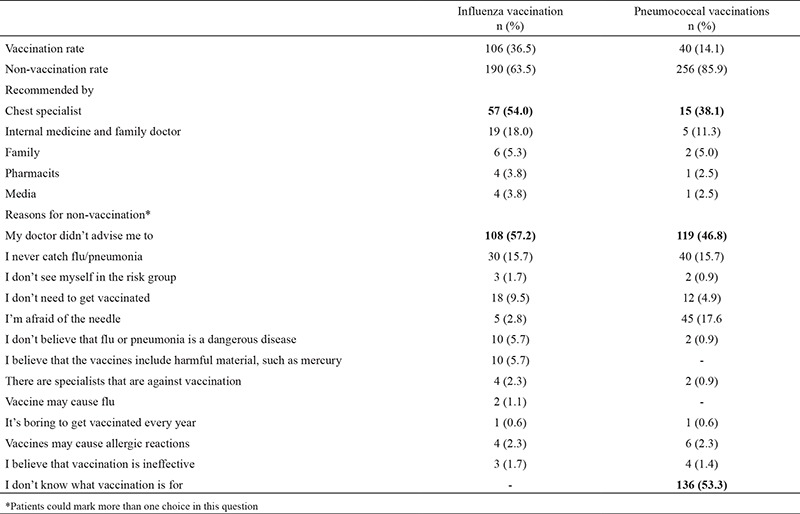
Vaccination situations of the patients

**Table 2:**
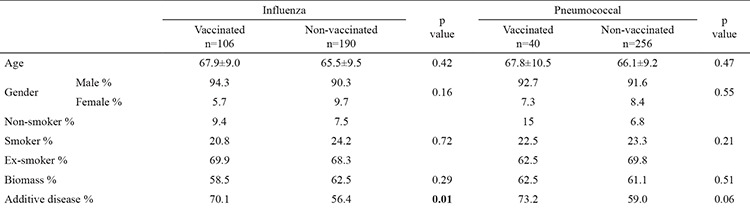
Demographic characteristics of the vaccinated and non-vaccinated patients

**Table 3:**
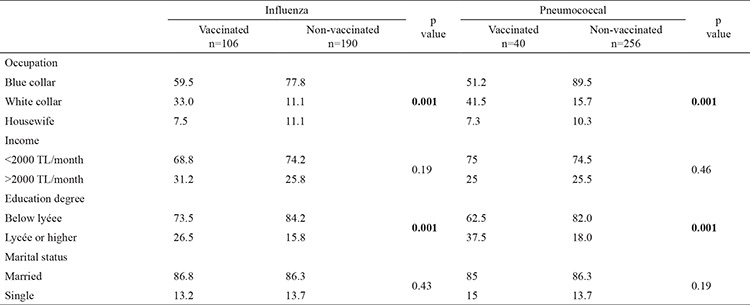
Socio-economic status of the vaccinated and non-vaccinated patients
